# Molecular Detection of *Toxoplasma gondii* and *Neospora caninum* in Domestic Ducks in Hunan Province, China

**DOI:** 10.3389/fvets.2021.649603

**Published:** 2021-04-15

**Authors:** Qiu-Yan Lv, He-Liang Zheng, Wen-He Yang, Guo-Hua Liu

**Affiliations:** ^1^Hunan Provincial Key Laboratory of Protein Engineering in Animal Vaccines, College of Veterinary Medicine, Hunan Agricultural University, Changsha, China; ^2^Hunan Co-Innovation Center of Animal Production Safety, Changsha, China

**Keywords:** *Toxoplasma gondii*, Neospora caninum, domestic ducks, PCR-RFLP, China

## Abstract

*Toxoplasma gondii* and *Neospora caninum* are protozoan parasites that infect warm-blooded animals, and cause major economic losses in livestock industries worldwide. However, little is known about the genotypes of *T. gondii* and *N. caninum* in domestic ducks in China. Herein, brain samples from 588 domestic ducks from Hunan province in China were examined for the presence of *T. gondii* and *N. caninum*. Polymerase chain reaction (PCR) was used to detect *T. gondii* B1 gene and *N. caninum NC-5* gene. Forty-five DNA samples (7.7%; 95% CI: 5.5–9.9) were positive for B1 gene, and two (0.3%; 95% CI: 0–0.7) were positive for NC-5 gene. The risk factors significantly associated with *T. gondii* infection were age and sex. The 45 samples positive for *T. gondii* were genotyped using multi-locus PCR-RFLP analysis and only one sample was fully genotyped as ToxoDB#9 (Chinese I). These results provide new information about the epidemiology of *T. gondii* and *N. caninum* in ducks in Hunan province in China. The data also highlight the importance of a “One Health” approach to dealing with toxoplasmosis.

## Introduction

*Toxoplasma gondii* and *Neospora caninum* are two important and highly prevalent protozoan parasites (1, 2). Toxoplasmosis, caused by *T. gondii*, is a widespread zoonotic disease causing significantly economic losses in animals and serious public health impacts on humans (3, 4). *T. gondii* in pregnant women may be transmitted to fetus and cause severe neurological sequelae (5, 6). Neosporosis, caused by *N. caninum*, is one of the most important causes of abortion in ruminants, particularly in cattle (7, 8). *N. caninum* is not considered a zoonotic parasite, but low antibody titers to *N. caninum* have been reported in humans (9–11).

Domestic cats and wild felids serve as definitive hosts of *T. gondii*, while dogs and wild canines play the role of definitive hosts of *N. caninum*. Other warm-blooded vertebrate animals (including birds) have been reported as intermediated hosts for both *T. gondii* and *N. caninum* (12–14). Various avian species play an important role in the life cycle of these parasites by serving as intermediate hosts. Avian species can be infected by *T. gondii* and *N. caninum* mainly via ingestion of sporulated oocysts from contaminated environments by feline and canine feces, respectively (15, 16). Domestic ducks serve as a common food source particularly in China. The per capita consumption of duck meat in China was 6.75 kg in 2019. Chinese people often eat undercooked duck meat as roast, spicy or dried. Additionally, duck blood in chili sauce (undercooked food) has recently become popular in many parts of China.

In China, ToxoDB#9 (also named as Chinese I) is the most common genotype in domestic animals, followed by ToxoDB#10 (17, 18). However, limited information is available concerning the molecular prevalence of *T. gondii* in domestic ducks in China. Only one study carried out by Zou et al. (19) showed that genotype ToxoDB#9 was predominant in poultry (including 115 duck meats) in Shandong province of China, indicating that the genetic variation of *T. gondii* in poultry in this province is limited. In addition, low antibody titers to *N. caninum* were found and *N. caninum* DNA was detected in wild waterfowl in Italy, which suggests that wild waterfowl is susceptible to *N. caninum* (20). Nonetheless, domestic ducks as natural intermediate host of *N. caninum* have not been reported.

The aim of the present study was to determine the molecular prevalence, risk factors and genotypes of *T. gondii* and *N. caninum* in domestic ducks intended for human consumption in Hunan province, China. The results provide a baseline for future surveillance and control programs of these parasites in ducks in China.

## Materials and Methods

### Sample Collection

From October 2018 to March 2020, 588 free-range ducks were purchased from food markets in five representative regions of Hunan province, China (Table 1). From each food market, ~5% of the slaughtered ducks were randomly sampled, where brain tissue was collected from each ducks and frozen at −20°C until assayed. Information about the geographic region, season, sex, and age of each duck was gathered.

**Table 1:** Prevalence and risk factors for *Toxoplasma gondii* infection in domestic ducks in Hunan province, China.

**Factor**	**Category**	**No. tested**	**No. positive**	**Prevalence (%) (95% CI)**	**Adjusted Odds ratio (95% CI)**	***P-*value**
Region	Eastern	78	2	2.6 (0–6.1)	Reference	
	Southern	153	14	9.2 (4.6–13.8)	3.8 (0.8–17.3)	>0.05
	Central	75	7	9.3 (2.7–15.9)	3.9 (0.8–19.5)	>0.05
	Western	131	15	11.5 (6.0–17.0)	4.9 (1.1–22.1)	<0.05
	Northern	151	7	4.6 (1.3–7.9)	1.8 (0.4–9.1)	>0.05
Season	Spring	84	2	2.4 (0–5.7)	Reference	
	Summer	186	18	9.7 (5.4–14.0)	4.4 (1.0–19.4)	<0.05
	Autumn	165	19	11.5 (6.6–16.4)	5.3 (1.2–23.5)	<0.05
	Winter	153	6	3.9 (0.8–7.0)	1.7 (0.3–8.5)	>0.05
Age	0<year≤1	89	2	2.2 (0–5.2)	Reference	
	1<years≤2	499	43	8.6 (6.1–11.1)	4.1 (1.0–17.2)	<0.05
Sex	Male	181	6	3.3 (0.7–6.0)	Reference	
Total	Female	407 588	39 45	9.6 (6.7–12.5) 7.7 (5.5–9.9)	3.1 (1.3–7.4)	<0.01

### DNA Extraction and PCR Amplification

Approximately 30 mg was obtained from each brain sample and total genomic DNA was extracted using a commercial kit (Wizard® SV Genomic DNA Purification System, Promega, Madison, USA) according to the manufacturer's directions. A semi-nested PCR was performed to detect *T. gondii* B1 gene (131 bp) as previously described (21). This gene target has been extensively used for detecting *T. gondii* infection in pigs, sheep, chicken, and other animals (22–25). Two primer pairs were used to amplify regions of the B1 gene of *T. gondii*: the outer primers B1-F1: 5′-GGAACTGCATCCGTTCATGAG-3′ and B1-R1: 5′-TCTTTAAAGCGTTCGTGGTC-3′; and inner primers B1-F2: 5′-TGCATAGGTTGCAGTCACTG-3′ and B1-R2: 5′-GGCGACCAATCTGCGAATACACC-3′. PCR product of 191 and 134 bp were obtained from first and second round of PCR reaction, respectively. PCR reactions (25 μl) included 2.5 μl DNA, 12.5 μl commercial premix PPP master mix, 0.1 μl each primers (0.1 mM) and 9.8 μl nuclease-free water. The amplification conditions included a 5 min of initial denaturation at 94°C, followed by 35 cycles of 94°C for 10 s (denaturation), 57°C for 10 s (annealing), 72°C for 30 s (extension), and a final extension step at 72°C for 5 min. The amplification condition for the secondary PCR was identical to the primary PCR, except that the annealing temperature was 63°C (21). Positive (GT1 strain) and negative (ultrapure H_2_O) controls were included in each assay.

The *N. caninum* NC-5 gene (328 bp) was amplified using PCR as previously described (26, 27), and by using reaction conditions and primers (Np21: GGGTGTGCGTCCAATCCTGTAAC; NP6: CTCGCCAGTCAACCTACGTCTTCT) described previously (28). The PCR amplification reaction included 3 μl of total DNA, 12.5 μl of commercial premix PPP master mix, 0.1 μl of each PCR prime (0.1 mM) and the remaining 25 μl reaction volume was topped up with nuclease-free water. The amplification conditions included 5 min initial denaturation at 94°C, followed by 40 cycles of amplification (40 s at 94°C, 40 s at 94°C, 40 s at 72°C and a final extension step at 72°C for 10 min. Positive (*N. caninum* NC-1 strain) and negative (ultrapure H_2_O) controls were included in each assay.

Each PCR product was examined on agarose gel (1%) electrophoresis to verify that they presented the expected bands of the target genes. The positive PCR products for the NC-5 gene were submitted to the Sangon Biotech Company (Shanghai, China) for DNA sequencing.

### Genetic Characterization of *T. gondii*

The B1 gene-positive samples were genotyped at 10 genetic markers (SAG1, SAG2 (5′+3′ SAG2, alter. SAG2), SAG3, BTUB, GRA6, c22-8, c29-2, L358, PK1, and Apico) using the multi-locus PCR-RFLP analysis as previously described (29) (Table 2). Eight reference *T. gondii* strains (GT1, PTG, CTG, MAS, TgCgCa1, TgCatBr5, TgCatBr64, and TgRsCr1) were included as controls as reported in previous studies (33, 34). The genotype was determined by comparing its multilocus pattern to the pattern of all genotypes present in ToxoDB (http://toxodb.org/toxo/) (35).

**Table 2:** Genotyping result of *Toxoplasma gondii* infection in domestic ducks in Hunan province, China.

**Isolate ID**	**Host**	**Tissue**	**Location**	**SAG1**	**5'+3' SAG2**	**Alternative SAG2**	**SAG3**	**BTUB**	**GRA6**	**c22-8**	**c29-2**	**L358**	**PK1**	**Apico**	**Genotype**	**References**
GT1	Goat		United States	I	I	I	I	I	I	I	I	I	I	I	Reference, Type I, ToxoDB #10	(29)
PTG	Sheep		United States	II/III	II	II	II	II	II	II	II	II	II	II	Reference, Type II, ToxoDB #1	(29)
CTG	Cat		United States	II/III	III	III	III	III	III	III	III	III	III	III	Reference, Type III, ToxoDB #2	(29)
MAS	Human		France	u-1^*^	I	II	III	III	III	u-1^*^	I	I	III	I	Reference, ToxoDB #17	(29)
TgCgCa1	Cougar		Canada	I	II	II	III	II	II	II	u-1^*^	I	u-2^*^	I	Reference, ToxoDB #66	(30)
TgCatBr5	Cat		Brazil	I	III	III	III	III	III	I	I	I	u-1^*^	I	Reference, ToxoDB #19	(31)
TgCatBr64	Cat		Brazil	I	I	u-1	III	III	III	u-1	I	III	III	I	Reference, ToxoDB #111	(31)
TgRsCr1	Toucan		Costa Rica	u-1	I	II	III	I	III	u-2	I	I	III	I	Reference, ToxoDB #52	(32)
**Sample #105**	**Duck**	**Brain**	**Hunan, China**	**u-1**	**II**	**II**	**III**	**III**	**II**	**II**	**III**	**II**	**II**	**I**	**ToxoDB #9**	**Present study**

* *u-1 and u-2 represent unique RFLP genotypes, respectively*.

### Statistical Analysis

The data were analyzed using SPSS 20.0 (IBM, Chicago, IL, USA). Multivariable mixed-effects logistic regression model was used to determine the relationship between the prevalence of *T. gondii* and various factors related to the ducks examined in the study, including the geographic region, the season of collection, age, and sex. Probability (*P*) value <0.05 was considered as statistical significance.

## Results

In this study, the overall prevalence of *T. gondii* in domestic ducks in Hunan province was 7.7% (95% CI: 5.5–9.9) (45/588). The prevalence of *T. gondii* infection in domestic ducks was 2.6%, 9.2, 9.3, 11.5, and 4.6% in Eastern, Southern, Central, Western, and Northern Hunan, respectively. However, there was no significant statistical difference in domestic ducks from different regions (*P* > 0.05) in Hunan province compared to Western region (*P* < 0.05) (Table 1). The prevalence of *T. gondii* infection in different seasons is shown in Table 1. The highest prevalence was found in Autumn (11.5%; 95%CI:6.6–16.4), followed by Summer (9.7%; 95%CI: 5.4–14.0) and Spring (2.4%; 95%CI: 0–5.7), and these differences were statistically significant (*P* < 0.05) compared to Winter. The prevalence of *T. gondii* in domestic ducks of 1<years≤2 (8.6%; 95% CI: 6.1–11.1) was higher than ducks of 0<year≤1 (2.2%; 95% CI: 0–5.2) (Table 1), and these differences were statistically significant (*P* < 0.05). Logistic regression analysis showed that ducks 1<years≤2 of age (OR: 4.1; 95% CI: 1.0–17.2) had four times higher risk of being positive compared with ducks ≤1 year old. As is shown in Table 1, female ducks (9.6%, 95% CI: 6.7–12.5) had a higher prevalence than male ducks (3.3%, 95% CI: 0.7–6.0), and these differences were statistically significant (*P* < 0.01). Logistic regression analysis showed that female ducks (OR: 3.1; 95% CI: 1.3–7.4) had three times higher risk of acquiring *T. gondii* infection compared with male ducks. In the present study, only one brain sample was genotyped at all loci, which was identified as genotype ToxoDB#9 (Table 2).

Two (0.3%; 95% CI: 0–0.7) of the 588 examined brain samples were positive for *N. caninum* Nc-5 gene. The sequences of the amplicons of both samples were deposited in GenBank (GenBank accession nos. MW194292 and MW194293). The Nc-5 gene sequences of *N. caninum* had 99% similarity to *N. caninum* sequence published previously (GenBank accession no. KU253799).

## Discussion

The prevalence (7.7%) of *T. gondii* in ducks in present study was higher than that reported in doves (*Zenaida macroura*) in American country (1%) (36); pigeon (*Columba livia*) in Iran (6.9%) (37); wild ducks in the Czech Republic (38); and poultry in Shandong (7.37%) (19). However, this prevalence was significantly lower than that detected in starlings (*Sturnus vulgaris*) (12.8%); chickens (*Gallus domesticus*) (15.5%) and sparrows (*Passer domesticus*) (26.5%) in Iran (37) and sparrows (*Passer domesticus*) in Brazil (17.5%) (39). These differences might be related to different avian species or different husbandry practices.

The results showed that ducks 1<years≤2 of age (OR: 4.1; 95% CI: 1.0–17.2) had four times higher risk of being positive compared with ducks ≤1 year-old, indicating that age may be a risk factor for *T. gondii* infection, in agreement with previous studies (40–43). Age is widely considered as a risk factor for high infection rates of *T. gondii* (44, 45). This might be attributed to increased frequency of exposure to the infectious *T. gondii* oocysts or the cumulative effect of the time period during which an animal can be exposed to the parasite (43, 46). The present study also showed that female ducks (OR: 3.1; 95% CI: 1.3–7.4) had three times higher risk of acquiring *T. gondii* infection compared with male ducks, suggesting that female ducks are more susceptible to *T. gondii* than male ducks (47).

In the present study, only one brain sample showed complete genotype at all loci, which was identified as genotype ToxoDB#9 (Table 2), which is consistent with that reported in ducks in a previous study in China (19). In addition, the remaining 44 B1-positive samples were amplified at only 3–5 loci, so have limited significance to reveal the level of genetic variation of *T. gondii*. To date, although different genotypes of *T. gondii* have been reported in domestic avian species worldwide (*e.g.*, ToxoDB#2, 9, 10, 26, 53, 114, 225, 227, 278, 281, 282) (19, 48–52), ToxoDB#9 is the prominent genotype in domestic poultry in China, and has been also frequently reported in other animals in China (53). A previous study (19) indicated that only one genotype (ToxoDB#9) was identified from domestic ducks, suggesting that the genetic variation of *T. gondii* may be relatively low in domestic ducks in China. However, further investigations including more domestic duck samples from other provinces of China are required to ascertain the full extent of *T. gondii* genotypes in domestic ducks.

Although previous studies showed that *N. caninum* DNA has been detected in domestic and wild poultry (20, 54), it was not detected in domestic ducks. The present study revealed a low molecular prevalence (0.3%) of *N. caninum* in domestic ducks, which is significantly lower than that reported in wild waterfowl (28.6%) (20) and chickens (4%) (54). Differences in *N. caninum* prevalence are likely attributed to differences in climates, husbandry practices, detection methods, or geographical origins. Our finding provided further evidence that domestic ducks are natural intermediate hosts *N. caninum*. Previous studies (55–57) have shown that poultry get infected with *T. gondii* through ingestion of sporulated oocysts from a contaminated soil. So, it is possible that domestic ducks become infected with *N. caninum* via the same route.

Humans become infected with *T. gondii* mainly via ingestion of raw or undercooked meat of infected animals (58). The present and previous (19) studies revealed the presence of *T. gondii* infection in domestic ducks in China, highlighting the potential threat to human health. According to the Ministry of Agriculture and Rural Affairs of China, 9,444,400 metric tons (about 70% of the global total) (59) of duck meat was produced and consumed in China in 2019. Duck meat including roast, spicy and dried duck meat is very popular among most Chinese. More importantly, pregnant women are encouraged to consume duck products (including duck blood) due to cultural habits. The risk of *T. gondii* infection in humans greatly increases by eating undercooked infected meat or meat products obtained from ducks. Therefore, adequate cooking of potentially infected duck meat is the safest way to ensure that tissue cysts are deactivated, thereby preventing infection. The results of the present study should assist the duck meat industry and local regulatory agencies to optimize interventions to improve the safety of duck products.

## Conclusion

The present study provided new data on the prevalence and risk factors of *T. gondii* infection in domestic ducks in Hunan province, China. To our knowledge, this is the first study focusing on *N. caninum* in domestic ducks in China. Future studies should consider studying histopathological changes and viability assessment of the parasites present in the duck tissues. Our findings provide a baseline for future surveillance and control of these parasites in ducks in China and reaffirm the importance of a “One Health” approach to dealing with toxoplasmosis.

## Data Availability Statement

The datasets presented in this study can be found in online repositories. The names of the repository/repositories and accession number(s) can be found at: GenBank and accessions MW194292 and MW194293.

## Ethics Statement

The study was approved by the Ethics Committee of Hunan Agricultural University (No. 43321503).

## Author Contributions

G-HL conceived and designed the study and critically revised the manuscript. Q-YL performed the experiments, analyzed the data, and drafted the manuscript. H-LZ and W-HY helped in the study design. All authors read and approved the final manuscript.

## Conflict of Interest

The authors declare that the research was conducted in the absence of any commercial or financial relationships that could be construed as a potential conflict of interest.
